# Past eight-year malaria data in Gedeo zone, southern Ethiopia: trend, reporting-quality, spatiotemporal distribution, and association with socio-demographic and meteorological variables

**DOI:** 10.1186/s12879-021-05783-8

**Published:** 2021-01-21

**Authors:** Eshetu Molla, Sinknesh Wolde Behaksra, Fitsum G. Tadesse, Sisay Dugassa, Endalamaw Gadisa, Hassen Mamo

**Affiliations:** 1 Armauer Hansen Research Institute, Addis Ababa, Ethiopia; 2 Department of Medical Laboratory Sciences, Dilla University, Dilla, Ethiopia; 3 Department of Microbial, Cellular and Molecular Biology, Addis Ababa University, Addis Ababa, Ethiopia; 4 Institute of Biotechnology, Addis Ababa University, Addis Ababa, Ethiopia; 5 Aklilu Lemma Institute of Pathobiology, Addis Ababa University, Addis Ababa, Ethiopia

**Keywords:** Malaria, Retrospective, Data quality, Spatiotemporal trend, Meteorological factors, Gedeo zone

## Abstract

**Background:**

Informed decision making is underlined by all tiers in the health system. Poor data record system coupled with under- (over)-reporting of malaria cases affects the country’s malaria elimination activities. Thus, malaria data at health facilities and health offices are important particularly to monitor and evaluate the elimination progresses. This study was intended to assess overall reported malaria cases, reporting quality, spatiotemporal trends and factors associated in Gedeo zone, South Ethiopia.

**Methods:**

Past 8 years retrospective data stored in 17 health centers and 5 district health offices in Gedeo Zone, South Ethiopia were extracted. Malaria cases data at each health center with sociodemographic information, between January 2012 and December 2019, were included. Meteorological data were obtained from the national meteorology agency of Ethiopia. The data were analyzed using Stata 13.

**Results:**

A total of 485,414 suspected cases were examined for malaria during the previous 8 years at health centers. Of these suspects, 57,228 (11.79%) were confirmed malaria cases with an overall decline during the 8-year period. We noted that 3758 suspected cases and 467 confirmed malaria cases were not captured at the health offices. Based on the health centers records, the proportions of *Plasmodium falciparum* (49.74%) and *P. vivax* (47.59%) infection were nearly equivalent (*p* = 0.795). The former was higher at low altitudes while the latter was higher at higher altitudes. The over 15 years of age group accounted for 11.47% of confirmed malaria cases (*p* < 0.001). There was high spatiotemporal variation: the highest case record was during *Belg* (12.52%) and in Dilla town (18,150, 13.17%, *p* < 0.001) which is located at low altitude. Monthly rainfall and minimum temperature exhibited strong associations with confirmed malaria cases.

**Conclusion:**

A notable overall decline in malaria cases was observed during the eight-year period. Both *P. falciparum* and *P. vivax* were found at equivalent endemicity level; hence control measures should continue targeting both species. The noticed under reporting, the high malaria burden in urban settings, low altitudes and *Belg* season need spatiotemporal consideration by the elimination program.

**Supplementary Information:**

The online version contains supplementary material available at 10.1186/s12879-021-05783-8.

## Background

In Ethiopia, *Plasmodium falciparum* (*P. falciparum*) and *P. vivax* are the predominant causative species of malaria. The two coexist in almost all malarious areas at different levels of co-endemicity. Overall, large proportion of infections reported is due to *P. falciparum* (~ 60%) followed by *P. vivax* (~ 40%) [1, 2] with micro-epidemiological and seasonal variation. Such co-endemicity makes malaria control and elimination more complicated in Ethiopia than in most other areas where the later species is absent or very low [2, 3].

Malaria transmission in Ethiopia is seasonal associated with precipitation and temperature changes; peaking from September to December following the large rainy season from June to August in most parts of the country [4]. This rainfall pattern doesn’t include the southern and south-eastern parts of the country, which have a bimodal rainfall periods with long rainy season from March to May and short period from September to October [5]. However, construction of dams and irrigation-based agricultural activities sometimes modify malaria seasonal trend in Ethiopia [6, 7].

Human mobility/displacement is another contributing factor to the resurgence/distribution of infectious diseases such as malaria [8]. As some other parts of the country, there are also internal displacements in the study area. Among the possible reasons for displacements in the area include seasonal agricultural work especially for coffee cultivation and inter-communal conflict along the borders. Such human activities might have implication on the malaria distribution or reporting system and influence on services rendered at health facilities (or on the health system) in the area.

The past decades witnessed a sharp decline in morbidity and mortality, putting Ethiopia among the few African countries on track to meet the global 2020 milestone of cutting incidence by 40% or more [9]. These successes, enabled the Ethiopian national malaria control program (NMCP) to stratify the country’s malaria transmission into four based on annual parasite incidence (API); malaria free (API ~ 0 cases/1000 population/year), low (API > 0 and < 5), moderate (API ≥ 5 and < 100) and high (API ≥ 100) [4], as a preparation to embark on nationwide malaria elimination. The policy and strategy shift to elimination requires data-driven decision making to tailor interventions [10, 11]. Thus, policy makers should be provided timely with quality and relevant data to inform national programs.

In Ethiopia, malaria data is captured through the Public Health Emergency Management (PHEM) at different tiers of the healthcare delivery systems. The hierarchy of data flow is from health posts (HPs) and health centers (HCs) to district health offices (HOs) which in turn channels to Zonal Health Departments, then to Regional Health Bureaus and finally to the Federal Ministry of Health [12]. As malaria in Ethiopia is a weekly reportable disease, HCs are expected to report to the district HO gets registered through the PHEM system. Therefore, HCs data that are organized and archived at respective district HOs are the ones that are analyzed to evaluate the spatial and temporal changes, local malaria dynamics and *Plasmodia* species distribution.

Both internal and external data quality control system were there at the HCs level in the study setting. The internal quality control system was done by using different readings and smear preparations. External microscopy quality assessment was employed through sending the randomly selected, both positive and negative, slides to the regional reference laboratory.

Although the overall malaria trend could help to evaluate the progress of elimination activities, under- (over)-reporting of cases could pause a serious repercussion on the country’s elimination efforts. Yet, validation studies comparing data from the different tiers of the health care delivery system hardly exist in most settings and at micro-epidemiological level. Although the six malarious districts of Gedeo Zone are stratified as elimination targeted low transmission districts by NMCP [4], little information is available to understand the overall trend of malaria and the above issues in the area. Thus, we assessed the overall trend of malaria, species composition, malaria data quality, spatiotemporal distribution and associated socio-demographic and climatic variables. Further, the accuracy of HO malaria records (PHEM data) was checked against the HC data (source document).

## Methods

### Study setting

**Fig. 1:**
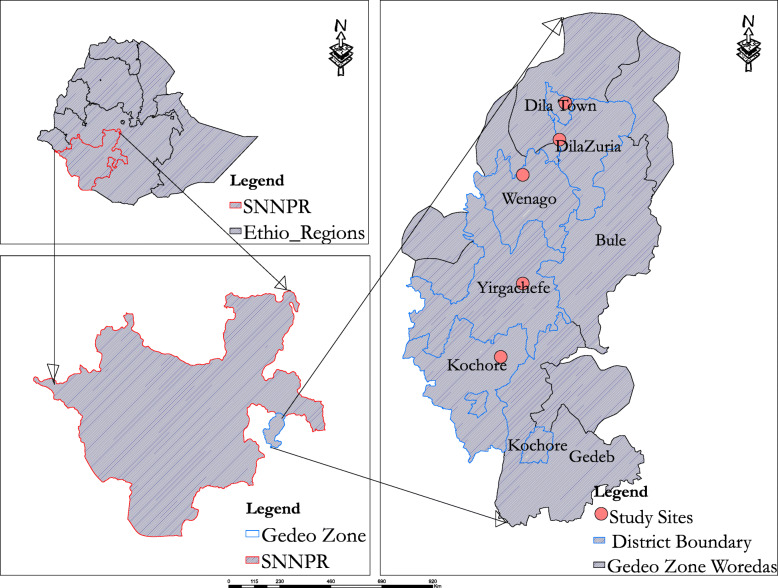
Map of the study districts; Gedeo Zone Southern Nationals Nationalities and Peoples Regional state (SNNPR), Ethiopia. Map produced by the authors using Arc-GIS Desktop v10.5 (Esri, Redlands CA, USA; https://www.redlands.edu/study/schools-and-centers/css/resources/arcgis-desktop/)

Gedeo zone (Fig. 1) is 360 km from Addis Ababa, it is one of the 14 zones in the Southern Nations, Nationalities and Peoples’ Region. It is located 5°53′N to 6°27′N latitude, and 38°8′ to 38°30′ east longitude. The altitude of the zone ranges from 1268 to 2993 m above sea level (masl). The mean annual temperature is between 12.6 °C and 30 °C and the mean annual rainfall ranges from 1001 to 1800 mm.

Based on the Gedeo Zone health department report, 36.31% (423,411/1,166,163) of the population is at risk of malaria. Malaria transmission in the zone is seasonal with peak from September to November. The API of the zone in 2019 was close to 2.0. The public health facilities in the area include; 1 referral hospital, 3 primary hospitals, 35 HCs and 146 HPs. The zone is sub-divide into six districts and two town administrations [13]. Districts, also known as “*woredas*” in Ethiopia, are the third level administrative divisions of the country, following regional states and zones and are further sub-divided into “*kebeles*” (the smallest administrative unit with its own jurisdiction). Six elimination-targeted settings, low transmission, by the NMCP; Dilla and Yirgacheffe towns, and Dilla zuria, Wonago, Kochore and Yirgacheffe rural districts were included [4]. Based on their altitudinal location, the study districts are within low altitude (< 1750 masl) and high altitude (1750–2500 masl) areas.

There are 3 categories of seasons in Ethiopia in general and Gedeo Zone in particular based on seasonal rainfall cycle. These are locally known as *Belg* (extends from February to May), *Kiremt* (June to September) and *Bega* (from October to January). *Belg* is the main rainy season for the south and southeast parts of Ethiopia including the current study area. But it is the minor rainy season in most parts of the country. *Kiremt* is the major rainy season across much of the nation except south and southeast of the country. It is the short rainy season in the area. Whereas, *Bega* (the dry-spell) is characterized by sunny, dry and cold weather condition with occasional falls prevailed over much of the country [5, 14]. As the present study area lies at the southern portions of Ethiopia, it benefited from a bimodal rainfall season with long periods during *Belg* and short rains from September to October [5].

### Data collection

Retrospective record review of malaria data from district HOs and HCs over eight-year period (January 2012–December 2019) in Gedeo zone, South Ethiopia, were extracted. Malaria laboratory registration logbooks in the HCs and PHEM of the district HOs were used to extract the data. HCs found in malarious districts with at least 8 years of services and HOs in these districts were considered as eligible for the study. Accordingly, six district HOs and 17 public HCs were covered. HC records with missing information of cases, address (*kebele*/district), dates of HC visit, age, sex or results of malaria diagnosis were excluded from the main part analysis. These excluded data were again analyzed separately to address the data quality issues at HCs. Data on malaria diagnosis results (negative, or positive, and infecting *Plasmodium* species for positives), time of diagnoses (date/month/year) and socio-demographic data (*kebele*/district, age and sex) were collected. Microscopy was used as confirmatory diagnostic tests in all HCs. In addition, 8-year meteorological data; station level monthly and annual precipitation (in mm), maximum and minimum temperatures (in ^o^C) and relative humidity (percent) was obtained from the national meteorology agency (NMA) of Ethiopia. The altitude records of each district were also collected using single hand-held Global Positioning System (GPS) (GPSMAP 64 s, 3BP329982). Data collectors attended adequate training to assure quality. Further, the consistency and completeness of the extracted data was checked for each HC and district. The completeness of data was checked by inspecting whether the completeness of variables for analysis, such as *Plasmodia* spp. case record, date, age, sex and address, from the captured data. The data is considered as complete when all variables needed for analysis are recorded. The consistency was again checked by whether the availability/matching of important variables and examination results at time (dates/months/years) and place (districts/HCs).

### Data analysis

Microsoft office excel worksheet 2019 and the Stata data software 13 (College Station, Texas 77845 USA) were used for data entry and analysis. Descriptive statistics was used to show the distribution of malaria cases with respect to months, years, sex, age, *Plasmodia* species and district. The proportion of *P. falciparum* and *P. vivax* was expressed as the total number of each species per total number of confirmed malaria cases. Pearson’s chi-square (*X*^*2*^) test was used to assess the data quality issues in terms of missing data (data incompleteness), consistency and diagnostic performance issues. Multivariate logistic regression was also performed to assess the association of malaria cases with socio-demographic variables, altitude and districts. Odds ratio (OR) with the corresponding 95% confidence interval (CI) was used to assess the differences in malaria prevalence with selected predictors. The generalized linear model (GLM) was used to investigate the association of monthly malaria cases (as response variable) with seasonal and meteorological data (as predictor variables). *P*-value of less than 0.05 was taken as statistically significant.

## Results

### Annual suspected and confirmed malaria cases based on HCs versus HOs records

Overall, a notable decline in malaria, HCs (source data), was observed during the eight-year period except in 2016 and between 2018 and 2019. The number of confirmed malaria cases declined from 11,607 in 2012 to 2857 in 2019, an 8.34% reduction from the baseline. Maximum and minimum numbers of confirmed cases were documented during 2013 and 2018 respectively. The case burden due to *P. falciparum* (49.74%) was comparable to *P. vivax* (47.59%) (*p* = 0.795). Yet, *P. vivax* overtook *P. falciparum* in cases burden during 2013, 2014 and 2017. Mixed species infections, *P. vivax* and *P. falciparum*, accounted a low proportion (2.67%) (Table 1).

**Table 1:** Numbers of suspected cases (N) and confirmed malaria cases (n) by year; HCs and HOs data, Gedeo zone, South Ethiopia, 2012–2019

Year	Data from HCs					Data from HOs			***P***-value
	Suspected cases (N)	Confirmed				Suspected cases (N)	Confirmed		
		***Pf*** n (%)	***Pv*** n (%)	***Pf***/***Pv*** mixed n (%)	***P***-value		***Pf*** n (%)	***Pv*** n (%)	
2012	80,188	5921 (51.01)	5403 (46.55)	283 (2.44)	**0.795**	80,188	5785 (48.40)	6168 (51.60)	**0.041**
2013	89,659	7386 (46.06)	8168 (50.93)	483 (3.01)		89,298	7876 (49.48)	8041 (50.52)	
2014	36,765	2110 (47.85)	2199 (49.86)	101 (2.29)		36,649	2277 (52.13)	2091 (47.87)	
2015	58,564	2642 (52.65)	2232 (44.48)	144 (2.87)		58,455	2289 (46.52)	2631 (53.48)	
2016	74,511	5660 (54.61)	4450 (42.93)	255 (2.46)		71,502	4937 (49.55)	5027 (50.45)	
2017	61,209	2014 (45.90)	2242 (51.09)	132 (3.01)		61,049	1928 (45.18)	2339 (54.82)	
2018	37,897	1298 (50.98)	1182 (46.43)	66 (2.59)		37,897	1313 (51.47)	1238 (48.53)	
2019	46,621	1437 (50.30)	1359 (47.57)	61 (2.13)		46,618	1442 (51.12)	1379 (48.88)	
**Total**	**485,414**	**28,468 (49.74)**	**27,235 (47.59)**	**1525 (2.67)**		**481,656**	**27,847 (49.06)**	**28,914 (50.94)**	

*N, n* number of cases*, %* percentage (n/N*100)*, Pf Plasmodium falciparum, Pv Plasmodium vivax*

Over the 8-years period (2012–2019), there is an evidence of statistically significant inconsistency (*p* = 0.041) in both clinical and confirmed malaria case reports between HCs and HOs, reporting different figures. Higher suspected cases (485,414) were recorded at HCs compared to the HOs (481,656). Similarly, the corresponding confirmed malaria cases were 57,228 (11.79%) at the HCs and 56,761 (11.78%) at HOs although establishing which one is more accurate is rather not easy. With this, the number of suspected cases recorded by the HCs was higher by 3758. The data kept by the HOs was lower on average by about 470 each year (except 2012, 2018). This difference was pronounced in 2016. During 2012 and 2018 the numbers of suspected cases recorded at HCs were consistent with HOs data. In addition, the PHEM captured on average 58 less confirmed malaria cases each year except in 2012 and 2018. In 2012 and 2018 the numbers of confirmed cases were higher at HOs records than at HCs records (Table 1).

### Year-based data with missing variables at HCs

In respective order 1852 (0.38%) and 249 (0.43%) of suspected cases and confirmed malaria cases were excluded from the downstream analysis due to incompleteness. Overall, there was a significant reduction in missing data between 2012 and 2019 (*p* = 0.001). During the first 2 years there were more numbers of both suspected and confirmed cases of missing variables. Specifically, the highest data with missing variables (413 suspected and 76 confirmed cases) occurred in 2012. Whereas, the lowest records of missing variables with 107 suspected and 10 confirmed cases were reported in 2019 and 2018 respectively. Generally, the number of suspected cases with missing variable declined from 413 in 2012 to 107 in 2019 by approximately 4-fold and confirmed cases from 76 in 2012 to 11 by 7-fold in 2019 (Table 2).

**Table 2:** Number of missing data on suspected cases and confirmed malaria cases by year, Gedeo zone, South Ethiopia, 2012–2019

Year	Grand total suspected cases	Excluded suspected cases (%)	Grand total confirmed cases	Excluded confirmed cases (%)	***P***-value
2012	80,601	413 (0.51)	11,683	76 (0.65)	0.001
2013	89,979	320 (0.36)	16,075	38 (0.24)	
2014	37,076	224 (0.60)	4450	40 (0.90)	
2015	58,788	311 (0.53)	5042	24 (0.48)	
2016	74,693	182 (0.24)	10,384	19 (0.18)	
2017	61,395	186 (0.30)	4419	31 (0.70)	
2018	38,006	109 (0.29)	2557	10 (0.39)	
2019	46,728	107 (0.23)	2867	11 (0.38)	
**Total**	**487,266**	**1852 (0.38)**	**57,477**	**249 (0.43)**	

*Grand total* quantitatively analyzed + excluded data (missing data that analyzed separately)

### Diagnostic performance; data from HCs

Regarding the diagnostic performance, the yearly trend of suspected cases was directly proportional to the confirmed cases in each year. But the proportions of examined suspected cases against confirmed cases were significantly increased from 6.91 in 2012 to 16.32 in 2019 (*p* = 0.015). The highest proportion (16.32) was recorded in 2019, whilst the lowest (5.59) was during 2013. This shows the proportion of ‘non-malarial’ febrile cases were increasing from 2012 to 2019 except in 2016 (Table 3).

**Table 3:** Proportion of suspected cases against confirmed malaria cases, Gedeo zone, South Ethiopia, 2012–2019

Year	Suspected cases (N)	Confirmed (n)	Proportion (N/n)	***P***-value
2012	80,188	11,607	6.91	0.015
2013	89,659	16,037	5.59	
2014	36,765	4410	8.34	
2015	58,564	5018	11.67	
2016	74,511	10,365	7.19	
2017	61,209	4388	13.95	
2018	37,897	2546	14.88	
2019	46,621	2857	16.32	
**Total**	**485,414**	**57,228**	**8.48**	

### Malaria cases number by sex and age; data from HCs

**Fig. 2:**
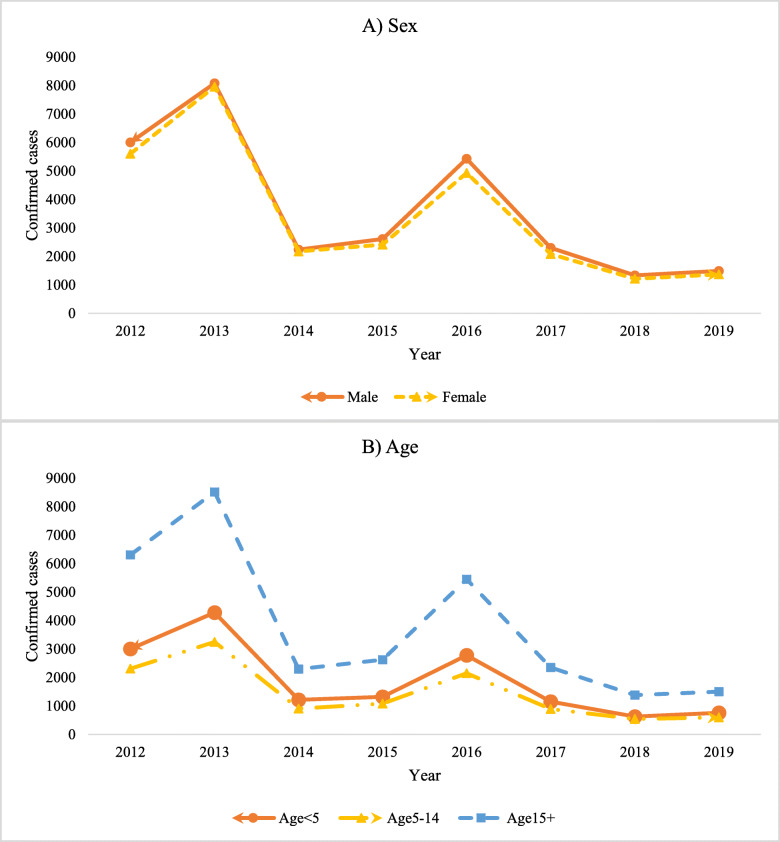
Annual clinical and confirmed malaria trend by sex and age in Gedeo zone, South Ethiopia from 2012 to 2019

Slightly more males (29,480 (11.34%)) were malaria positive (*p* > 0.05) than females (27,748 (12.30%)) (Fig. 2). The above 15 years age group was the most affected (30,406, 11.47%) than the other age groups, followed by under 5 children which accounted for 15,116 (13.83%) of the cases. The above 15 years age group was twice more likely (AOR = 2.00, 95% CI: 1.90, 2.11, *p* < 0.001) to have malaria compared to the under 5 (Table 4).

**Table 4:** Multivariate logistic regression analysis of factors associated with malaria at HCs in Gedeo zone, South Ethiopia, 2012–2019

Variable	Suspected cases N (%)	Confirmed n (%)	COR (95% CI)	AOR (95% CI)
**Sex**				
Male	259,906 (53.54)	29,480 (11.34)	1.0	1.0
Female	225,508 (46.46)	27,748 (12.30)	0.69 (0.73, 1.02)	1.02 (0.97, 1.08)
**Age category**				
< 5	109,302 (22.52)	15,116 (13.83)	1.0	1.0
5–14	111,028 (22.87)	11,706 (10.54)	1.53 (1.42, 1.77)^**^	1.33 (1.29, 1.37)^**^
15+	265,084 (54.61)	30,406 (11.47)	2.31 (2.19, 2.61)^**^	2.00 (1.90, 2.11)^**^
**District/urban center**				
Yirgacheffe rural^H^	49,684	5187 (10.44)	1.0	1.0
Dilla town^L^	137,860	18,150 (13.17)	4.87 (3.55, 6.02)^**^	3.16 (2.11, 4.22)^**^
Dilla zuria^L^	119,207	12,588 (10.56)	2.56 (2.17, 2.73)^**^	2.30 (1.82, 2.78)^**^
Wonago^H^	71,689	7427 (10.36)	1.86 (1.72, 1.95)^*^	1.40 (1.21, 1.60)^*^
Yirgacheffe town^H^	60,531	6271 (10.36)	0.33 (0.09, 1.65)	0.24 (0.07, 1.42)
Kochore^H^	46,443	7605 (16.37)	1.45 (1.14, 1.69)^**^	1.59 (1.20, 1.98)^**^

% of confirmed: percentage (n/N*100)

^**L**^: districts at low altitude (< 1750 masl); ^**H**^: districts at high altitude (1750–2500 masl)

*significant difference of *p* < 0.05, **significant difference at *p* < 0.001

### Spatiotemporal distribution of malaria cases

**Fig. 3:**
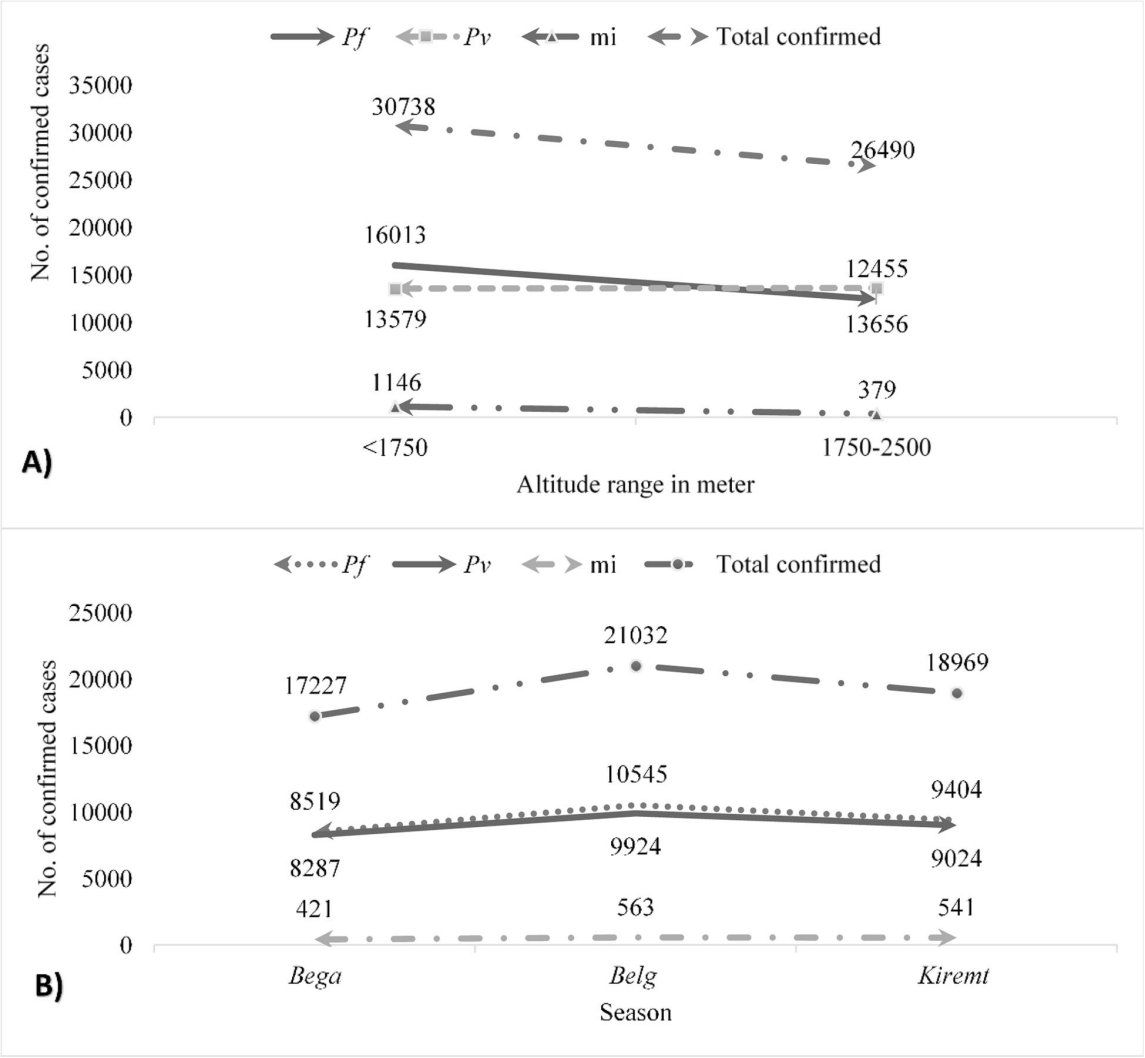
The malaria parasite distribution with **a** altitude and **b** season based on HC data, Gedeo zone, South Ethiopia, 2012–2019

Overall confirmed malaria cases were significantly influenced by altitude, the higher in districts of < 1750 masl (53.71%). People residing in districts < 1750 masl were 1.2 times more likely to be infected with malaria as compared to those living in districts between 1750 and 2500 masl (AOR = 1.22, 95% CI: 1.12, 1.29). *Plasmodium vivax* (13656) was relatively higher at higher altitudes (1750–2500 masl) while *P. falciparum* (16013) was higher at the lower altitudes (< 1750 masl). But elevation and species distribution showed no statistically significant associations (Fig. 3a).

The peak confirmed case load (12.52%) was during *Belg* followed by *Kiremt* (11.28%) and *Bega* (11.55%). In *Belg,* the peak transmission season in the study area*,* the likelihood of having malaria is 1.28 times more than *Bega* (RR = 1.28, 95% CI: 1.09, 1.48). Similarly, the highest proportions of both *P. falciparum* (10,545 (50.14%)) and *P. vivax* (9924 (47.19%)) were noted during *Belg* particularly in April. On the other hand, in *Bega* the proportion of infection due to the two species, *P. falciparum* (8519, 49.45%) and *P. vivax* (8287, 48.11%) was relatively lowest. Although *P. falciparum* was higher than *P. vivax* in all seasons, the difference was the smallest (1.35%) during *Bega*, while in *Belg* it is 2.95%. The number of mixed infections also had same pattern throughout the three seasons (Fig. 3b).

**Fig. 4:**
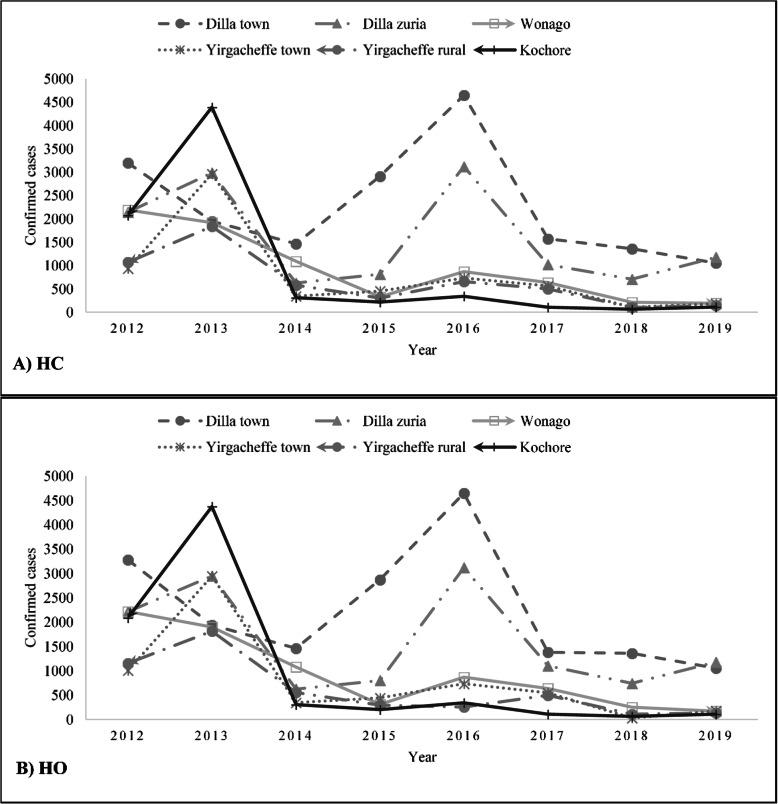
Year-based spatial distribution of confirmed malaria cases in HCs and HOs, Gedeo zone, South Ethiopia, 2012–2019

Overall, based on the HCs records, the highest malaria case burden reported was from Dilla town (18,150 (13.17%)) from a total of 137,860 suspected cases followed by the adjacent district, Dilla zuria (12,588 (10.56%)) from 119,207 tested suspects. Both of the districts are < 1750 masl. The lowest was from Yirgacheffe rural district (5187 (10.44%), at 1750–2500 masl). Dilla town annual malaria cases remained the highest throughout the 8-year period except in 2013 and 2019 (AOR = 3.16, 95% CI: 2.11, 4.22, *p* < 0.001) (Table 4). The highest confirmed cases during these 2 years were reported by Kochore (4390, 19.78%) and Dilla zuria districts (1178, 8.04%) from 22,197 and 14,648 suspected cases respectively. Although there was an overall declining trend of confirmed malaria cases from 2012 to 2019, there were increases in Kochore during 2013, Dilla town and Dilla zuria in 2016 (Fig. 4). *Plasmodia* spp. distribution varied with district, the highest number of *P. falciparum* cases was seen in Yirgacheffe and Dilla towns and Dilla zuria district while *P. vivax* overtook in other districts (Supplementary Fig. 1).

Yirgacheffe rural district had the highest variation (376, 80.51%) in terms of confirmed malaria records between HC and HO data (HC-HO) followed by Dilla town (164, 35.12%) and Dilla zuria district (− 138, 29.55%), data obtained by HCs-HOs. Whereas, Wonago district had the lowest (− 6, 1.28%) inconsistency in malaria data recordings between HCs and HOs (Fig. 4).

**Fig. 5:**
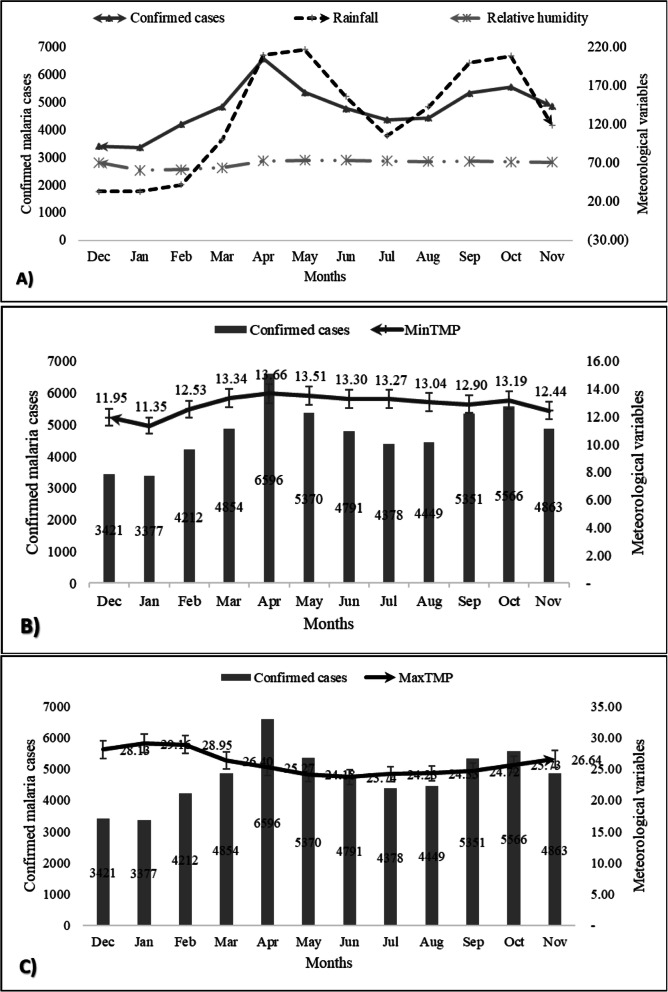
Monthly confirmed malaria cases with average **a** rainfall and humidity, **b** minimum temperature and **c** maximum temperature in Gedeo zone, South Ethiopia from 2012 to 2019

The monthly average confirmed malaria cases showed a strong association with monthly rainfall (RR = 11.47, 95% CI: 7.27, 15.68) and monthly minimum temperature (RR = 1.62, 95% CI: 1.05, 2.23) (Supplementary Table 1). Nevertheless, the maximum monthly temperature was negatively and weakly associated with the monthly confirmed malaria cases, as entire interval is below 1. Relative humidity (RH) seems also not to have a significant effect on malaria transmission in the study area. Apart from statistical association the lowest (60.8%) and highest (73.8%) records of RH were linked to monthly confirmed malaria cases. Relationship of monthly confirmed case records and climatic variables were displayed below (Fig. 5).

## Discussion

There was an overall reduction of malaria case from 2012 to 2019. According to data from HCs (source data), a maximum of 16,037 and a minimum of 2546 of cases were observed during 2013 and 2018, respectively with 8.34% reduction. 2013 and 2016 were the exceptions to the declining trend as there were case increases in these periods in some parts of the Zone. The relatively lower malaria cases for the periods between 2014 and 2015 could be associated with rainfall variations and strong intervention activities in the zone. 2016 was an El Nino year that predominantly affected the normal rainfall frequency and distribution along the southern Ethiopia. Thus, this could be the possible contributing factor for the higher occurrence of malaria in the area during 2016. The overall malaria positivity rate (11.79%) in the current study was comparable with certain studies done in Ethiopia including from Batu town (12.43%) [15], Halaba special district (9.47%) [16] and north Shoa (8.39%) [17]. In contrast, higher overall malaria positivity rates were reported from related studies conducted in south-central Ethiopia [18], southern Ethiopia [19] and abroad in Dakar, Senegal [20] with 33.83, 21.79 and 19.68% respectively. On the other hand, the present figure was higher than records in other local studies [21, 22]. These differences might be due to the variation in quality of laboratory diagnoses, difference in intervention measures, micro-climatic/altitudinal differences, and presence of constructions responsible for occurrence of temporary and permanent dams and drug and insecticide resistances.

*P. falciparum* and *P. vivax* were detected where equivalent; congruent results were reported in some other parts of Ethiopia [18, 19]. While other local studies [15, 17, 23] documented that the dominant species was *P. vivax*. The proportion of mixed infection in this study was congruent with other studies [18, 22], whereas inconsistent with other reports [19, 23]. The higher number of *P. vivax* against the national figures could also be an implication for the ability of repeated relapse cases and early emergence of gametocytes during blood-stage infection. In addition, there could be heterogeneity of the Duffy phenotype and the high number of vulnerable Duffy-positive individuals that associated with population movement [24] in the study area. Environmental fluctuations that change target mosquito species abundance might have an impact on *Plasmodia* species occurrence [25]. Further research is required to clarify whether the equivalent proportion of the two species is associated with the current practice/uptake of primaquine for *P. vivax* in the study area. The Ethiopian Federal Ministry of Health states that radical cure primaquine combined with chloroquine should be used to treat vivax malaria cases without prior G6PD testing [4]. The possible reason for scarcity of mixed infections in this co-endemic area might be a competitive or an antagonistic effect of one *Plasmodium* species over the other within the human host during co-infection [18, 25]. In addition, since the area is low transmission setting, mixed infections with *P. falciparum* and *P. vivax* infection are less likely to be detected by microscopy. This is because, *P. vivax* has an inherently lower parasitemia than its counterpart species, and thus less likely to be detected in a co-infection with *P. falciparum*.

For the 8-years period, there was an overall notable difference in both total number of suspected cases and confirmed malaria cases recorded at HCs (source data) and HOs (PHEM data). During 2012 and 2018 the numbers of suspected cases recorded at HCs were consistent with HOs data. In these years the numbers of confirmed cases were higher at HOs records than HCs records. Yirgacheffe rural district had the highest deviation in terms of confirmed malaria records between HCs and HOs data followed by Dilla town and Dilla zuria district, whereas Wonago district was the least. This inconsistency could affect the quality control system of the facilities in the zone. Similar to our finding, a three-month facility-based study comprising of various settings conducted in southern Ethiopia showed that majority of facilities under-reported total malaria (both confirmed and clinical malaria) cases [12]. Contrary to the present study, a study employed in three provinces of Mozambique stated that under-reporting of suspected and confirmed cases is a bigger issue than over-reporting in the health facility records as compared to the values reported through the health management information system (HMIS). They reported that values of both suspected and confirmed malaria cases documented at health facility records were lower than those reported through the HMIS [26]. This deviation in malaria data between the two systems, HC and HO, could be due to errors during entering the data from the sources (HCs) into recording formats of PHEM, lack of cross-checking and proofing habits, training gaps on PHEM data use and unintentional/intentional false reports. In addition, limited computer access and skill, inadequate technical support [27], poor data management skills and limited functionality of electronic data management systems [28] might be the likely reasons for PHEM implementation challenges.

In the present study there was a significant reduction in data incompleteness; with missing variables between 2012 and 2019 in terms of both suspected and confirmed cases. Apart from the statistical figures of declining trend of missing data (data incompleteness) from year-to-year, there are substantial proportions of missing data in recent years in the health facilities of the study area. When we ignored the missing data there could be over/under estimates of malaria data affecting the real figure of API [29] in the area. Since API estimates are vital for malaria stratification and further elimination activities. Thus, the declining trends of data incompleteness noted in this study needs to be sustained as the country’s NMCP has prioritized improving data quality issue in its strategic plan.

Concerning the diagnostic performance, the proportions of suspected cases against confirmed cases were increased from year to year. The decreased proportion of confirmed malaria cases detected could likely be due to decreasing malaria incidence in the study area. But the proportions of ‘non-malarial’ febrile cases were increasing from 2012 to 2019. This can be an indication of declining lab capacity of detecting malaria parasites. This declining lab capacity of detecting malaria might be due to the fact that the increased false negative reports associated with reduced sensitivity of microscopy with decreasing parasite densities [30], unable to detect sequestered *P. falciparum* parasites [31] and low competency of microscopists [32]. In the other way, such increased number of non-malarial febrile illnesses might be related to other febrile cases including yellow fever virus [33, 34] and typhoid fever [35, 36] infections, as per the studies conducted in southern and south-central Ethiopia. In addition, this high number of non-malarial febrile illness might be due to fevers among positive individuals with malaria where the fever is coexisted with but not caused by the *Plasmodia* infection [37]. Thus, non-malarial febrile illnesses of bacterial and viral (e.g. now covid-19) etiologies could complicate malaria diagnosis. This needs further investigation in the area. If laboratory performance percent confirmed declines it means; laboratory performance was decreasing over the years or something causing febrile illness in the area is increasing. Misdiagnosis and treating malaria clinically based only on fever with antimalarial drugs, which is still in remote settings, may contribute to the rapid emergence of antimalarial drug resistance [38, 39].

The slightly more infection among males in the present study was paralleled with other studies conducted in different parts of Ethiopia [15, 18, 19, 23]. However, this finding was not consistent with other reports in southern Ethiopia [17] and elsewhere in Mozambique [40] where higher malaria cases in females were documented. Individuals in the age group of 15 and above were also more significantly affected. This was in line with other local studies [15, 23]. In contrast, a finding in Metema, northwest Ethiopia by Ferede et al. [41] showed that 5–14 years old were more infected. Possible justifications for the higher occurrence of malaria among males and young adults and above age group could be their engagement in various outdoor activities and staying outdoors during the nights [42]. Apart from outdoor exposures, differences in treatment-seeking behavior, access to health facilities and travel history [43] might be the possible contributors for the sex- and age-based variations of malaria cases. In addition, a review report revealed that adult females are better protected from parasitic diseases than males due to genetic and biological (hormonal) factors [44].

Confirmed malaria cases were significantly influenced by altitude in the present study area. Higher malaria was in areas of low altitude (below 1750 masl) compared to in high altitudes (1750–2500 masl). *Plasmodia* spp. distribution also varied with elevation; *P. vivax* was relatively higher at higher altitudes while *P. falciparum* was higher at the lower altitudes. The likely reason for the slightly higher proportion of *P. falciparum* in lower altitudes and higher proportion of *P. vivax* in high elevations could be related to temperatures. That is, cooler environments are suitable for *P. vivax* while *P. falciparum* are adapted to a relatively higher temperature for the growth in human host and mosquito vectors [45, 46].

The peak number of confirmed malaria cases was recorded during *Belg*, following the long rainy season in the area, followed by *Kiremt*, short rainy season in the area, and *Bega*, the dry season in the area, with a statistically significant variation. The long rain season and peak malaria cases association seen in this finding is consistent with various studies in Ethiopia [17–19, 23] although the long rain season in these areas is from June to September. The high prevalence of malaria cases during the two seasons, following the rainfall pattern corroborates the two-season transmission pattern in the entire country. Following the long rainy season higher proportion of *P. falciparum* was recorded than *P. vivax*, while the difference was smallest during the dry season. The comparable proportion of *P. vivax* against *P. falciparum* in *Bega* might be explained by the fact that *P. vivax* has ability to relapse rather than new infections. Since such traits could affect the temporal patterns of *P. vivax* infections.

Overall, high number of suspected cases and confirmed malaria cases were documented in Dilla town (urban) and Dilla zuria district (sub-urban) which are found at lower elevations. Except in 2013 and 2019, Dilla town annual malaria cases remained the highest all over the 8-year period. The highest confirmed cases during these 2 years were overtaken by Kochore in 2013 and Dilla zuria districts in 2019. While the lowest was from Yirgacheffe rural district. In general, though an overall declining trend of confirmed malaria cases from 2012 to 2019, increments were recorded in Kochore during 2013 and Dilla town in 2016. Although there is expectation of a better documentation, treatment-seeking behavior, access to health facilities, community knowledge and coverage of intervention activities in urban settings, the current data pointed to the contrary. Thus, in this study, high burden of urban and suburban malaria was noted. This could be because of massive construction activities (like road, house and small dams) and presence of coffee processing sites in Dilla town and its vicinity that could create suitable habitat for mosquito breeding. Travel history [43], differences in the competence and skills of the laboratory personnel and relatively good reporting system might also be the main responsible factors influencing the prevalence of malaria in Dilla town compared to rural districts. There have been healthy ongoing malaria control activities incorporating environmental management, indoor residual spraying (IRS), long-lasting insecticide-treated nets (LLINs) and artemisinin-based combination therapy in the area. These intervention activities could be attributed for the decreasing trends of malaria in other sites of the Zone. In addition, micro-environmental variations, micro-climatic situations [25] and changes in intervention (like IRS and LLINs) periods might have effect for these spatial differences of malaria cases. The higher prevalence of malaria in districts with low altitudes would indicate the association of malaria and elevation.

Monthly rainfall and minimum temperature demonstrated statistically significant correlation with malaria cases. Previous studies in Ethiopia [47, 48] and elsewhere [49, 50] documented similar findings. This association suggests an early warning system can be developed to forecast the start of the malaria season using rainfall and temperature forecasts in the area. However, the result of the current study on the association of rainfall and malaria cases was deviating from previous finding, stating higher rainfall does not necessarily influence the malaria changes [51]. This deviation may be due to high rainfall affected the breeding sites of mosquito vectors in other places apart from the current study area [52]. In addition, there could be prevention practice variations between the population of the current study area and other sites in taking preventive measures during and after the high rain seasons. In contrast to our finding, minimum temperature was weakly correlated with malaria cases in southwest Ethiopia [48]. Ideally, rainfall and minimum temperature play a vital role in breeding and survival of malaria vectors and the respective parasites. Moreover, average monthly maximum temperature and RH were weakly correlated with malaria cases. In disagreement to our finding, studies conducted in Jimma, Ethiopia by Alemu and others [47] and Sena and colleagues [48] in Gilgel-Gibe, southwest Ethiopia reported that inter-monthly RH was significantly associated with monthly malaria cases. Publications of Akinbobola and colleague showed humidity levels between 60 and 90% were favorable for breeding and multiplication of *Plasmodia* parasites [53]. Although RH in our study was occurred between these risky ranges, no strong correlation with malaria cases was seen. This could be explained by the fact that RH is affected by rainfall and temperature, these might confound the relationship. Yet, additional research is required to explain the relationship between malaria transmission and RH in the area.

The limitation of this study was incompleteness of patient data in the register with missed variables and only 8-year data were available during the data collection time at the HCs. This study was limited to collecting secondary data only at health facility level and district level PHEM data. This prevented the readers for getting a clear situation how there is mismanagement of data at regional and federal levels in the country. Since the age classification on the laboratory registration books of HCs were only < 1, 1–4, 5–14, 15+, it’s difficult to further subdividing of 15+ age groups. Malaria diagnosis in the urban areas (particularly in Dilla and Yirgacheffe towns) could also be done in other public health facilities and private clinics. But this study did not include data collected from these facilities and this might underestimate the actual trend of malaria in the area. Furthermore, clinically treated patients’ (without laboratory confirmation) data and malaria mortality data were not recorded in the laboratory registration logbooks. Hence, interpretation of the finding should be with caution.

## Conclusion

Over the entire period, 2012 to 2019, the malaria control program performance improved; there was an overall drop in malaria burden and the proportion of data incompleteness declined significantly over the years. Yet, in the move for elimination data inconsistency between HCs and district HOs call for urgent correction. The equivalent *P. falciparum* and *P. vivax* co-endemicity level underline the need for interventions targeting both species. The high malaria prevalence in urban setting (Dilla town) and its vicinity requires consideration by the control campaign. The seasonal variation demands strengthening of interventions through short-term forecasting based on local meteorological factors.

## Supplementary Information

**Additional file 1:** **Supplementary Figure 1.** District level *Plasmodia* species distribution in Gedeo zone, South Ethiopia, 2012–2019. **Supplementary Table 1.** Association of HC-level monthly confirmed malaria cases and meteorological variables using GLM regression, Gedeo zone, 2012–2019.

## Data Availability

The datasets used and/or analyzed during the current study are available from the corresponding author on reasonable request.

## Abbreviations

*Pf*: *Plasmodium falciparum*
*Pv*: *Plasmodium vivax*
NMCP: National malaria control program
API: Annual parasite incidence
PHEM: Public Health Emergency Management
HP: Health post
HC: Health center
HO: Health office
masl: Meters above sea level
NMA: National meteorology agency
COR: Crude odds ratio
AOR: Adjusted odds ratio
CI: Confidence interval
GLM: Generalized linear model
RH: Relative humidity
HMIS: Health management information system
IRS: Indoor residual spraying
LLINs: Long-lasting insecticide-treated nets
