# Supplementary material for: Past eight-year malaria data in Gedeo zone, southern Ethiopia: trend, reporting-quality, spatiotemporal distribution, and association with socio-demographic and meteorological variables
Source: BMC Infect Dis. 2021 Jan 21;21:91. doi: 10.1186/s12879-021-05783-8 (PMC7817977; doi:10.1186/s12879-021-05783-8)
Supplement: Supplementary file 1 — Additional file 1: Supplementary Figure 1. District level Plasmodia species distribution in Gedeo zone, South Ethiopia, 2012–2019. Supplementary Table 1. Association of HC-level monthly confirmed malaria cases and meteorological variables using GLM regression, Gedeo zone, 2012–2019. [file 12879_2021_5783_MOESM1_ESM.doc]

**Supplementary file**s


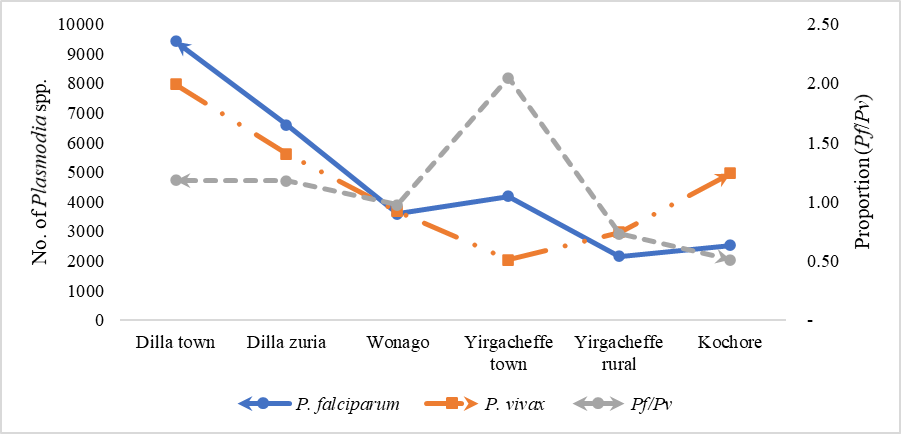


**Supplementary figure 1.** District level *Plasmodia* species distribution in Gedeo zone, South Ethiopia, 2012-2019

Supplementary table 1. Association of HC-level monthly confirmed malaria cases and meteorological variables using GLM regression, Gedeo zone, 2012-2019

| Meteorological factors | RR | 95% CI |
| --- | --- | --- |
| Monthly minimum temperature | 1.62 | 1.05, 2.23 |
| Monthly maximum temperature | 0.58 | 0.52, 0.66 |
| Monthly average rainfall | 11.47 | 7.27, 15.68 |
| Monthly average relative humidity | 1.17 | 0.63, 1.74 |

*RR: rate ratio*
